# Development of a ferritin-based subunit nanoparticle vaccine targeting the S-RBD of porcine transmissible gastroenteritis virus

**DOI:** 10.3389/fvets.2026.1805298

**Published:** 2026-03-19

**Authors:** Nannan Nie, Haoyu Yan, Li Zhang, Yingjuan Qian, Shanyuan Zhu, Yong-Sam Jung, Shinuo Cao

**Affiliations:** 1 Sanya Institute of Nanjing Agricultural University, Nanjing Agricultural University, Nanjing, China; 2 Jiangsu Key Laboratory for High-Tech Research and Development of Veterinary Biopharmaceuticals, Jiangsu Agri-animal Husbandry Vocational College, Taizhou, China

**Keywords:** ferritin, self-assembling nanoparticle, spike protein receptor-binding domain, subunit vaccine, TGEV

## Abstract

Porcine transmissible gastroenteritis virus (TGEV) remains a critical economic threat to the global swine industry due to its near 100% mortality rate in newborn piglets within 5 days of age; however, current vaccine strategies, such as attenuated vaccines, are often limited by biosafety concerns, whereas traditional inactivated vaccines, while resolving biosafety issues, exhibit poor immunogenicity. To address these limitations, this study aimed to develop a novel subunit vaccine by engineering self-assembling ferritin nanoparticles engineered to display the TGEV Spike protein receptor-binding domain (S-RBD). By fusing the S-RBD to the N-terminus of ferritin via a flexible linker, we generated eukaryotic expression plasmids and produced the recombinant proteins at scale using a lentiviral-transduced ExpiCHO cell system. Subsequent characterization via SDS-PAGE, Western blotting, and transmission electron microscopy (TEM) revealed that both TGEV-S-RBD-FR and ferritin scaffolds were successfully expressed at their predicted molecular weights of 72 kDa and 40 kDa, respectively. Critically, TEM and particle size analysis confirmed that these constructs assembled into monodisperse, spherical nanoparticles, with a diameter increase from 15 nm to 25 nm, validating the successful external display of the S-RBD. Collectively, these results demonstrate the successful development of a nanoparticle platform, offering a promising and highly programmable strategy for the prevention and control of TGEV.

## 1 Introduction

Porcine Transmissible Gastroenteritis Virus (TGEV) represents one of the most economically devastating enteric pathogens affecting the global swine industry (1, 2). As a member of the Alphacoronavirus genus, TGEV demonstrates a pronounced tropism for villous epithelial cells within the small intestine, inducing severe villous atrophy that compromises nutrient absorption and intestinal barrier function (3–5). The resulting clinical syndrome is characterized by an acute onset of vomiting, profuse watery diarrhea, and rapid dehydration (6). While adult pigs generally exhibit self-limiting disease with favorable recovery rates, the pathogenesis in neonatal piglets is markedly more severe, with mortality rates approaching 100% in animals under 2 weeks of age (7–10). This age-dependent susceptibility, combined with growth impairment in surviving animals, imposes substantial economic burdens on livestock producers and underscores the critical need for effective preventive strategies (11, 12).

The pathogenesis of TGEV is fundamentally dependent upon successful viral entry into target cells, a process mediated by the trimeric Spike (S) glycoprotein that forms the characteristic corona surrounding the viral envelope (13). Within each S protein monomer, the Receptor Binding Domain (RBD) constitutes the key functional determinant responsible for specific recognition and high-affinity binding to porcine aminopeptidase N (pAPN), the primary cellular receptor (14, 15). This molecular interaction represents the initial and rate-limiting step in viral pathogenesis, making the S-RBD an attractive target for therapeutic intervention (16, 17). The strategic importance of the S-RBD extends beyond its role in viral entry; this domain harbors the majority of conformational epitopes recognized by neutralizing antibodies capable of blocking virus-receptor interactions. Consequently, the S-RBD is currently an antigenic target with great advantages for subunit vaccine development, offering the potential to elicit protective humoral immunity while avoiding the safety concerns associated with whole-virus preparations (18).

Despite this clear molecular target, current TGEV control strategies remain heavily dependent on conventional vaccine platforms that exhibit significant limitations (19–22). Live-attenuated vaccines, while capable of inducing robust and durable immunity, carry inherent biosafety risks including potential reversion to virulence and genetic recombination with circulating wild-type strains (23). Inactivated vaccines circumvent these safety concerns but typically demonstrate suboptimal immunogenicity, particularly in their inability to stimulate adequate mucosal immune responses essential for protection against enteric pathogens (24, 25). Additionally, both platforms frequently require multiple booster administrations due to waning immunity, increasing both cost and complexity of vaccination protocols (26). These persistent limitations necessitate the development of next-generation vaccine technologies that can overcome the inherent trade-offs between safety and efficacy.

Self-assembling protein nanoparticles represent a paradigm shift in rational vaccine design, offering unique advantages in antigen presentation and immunological recognition (18, 27). Ferritin, an evolutionarily conserved iron-storage protein found across diverse species, exemplifies this approach through its intrinsic capacity for spontaneous assembly into highly stable, spherical 24-mer nanoparticles with icosahedral symmetry (28). The genetic fusion of heterologous antigens to the exterior surface of Ferritin subunits results in multivalent antigen display that closely mimics the repetitive, organized structure characteristic of viral particles. This biomimetic architecture enhances antigen recognition by B-cell receptors, promotes efficient antigen trafficking to germinal centers, and stimulates robust humoral immune responses that significantly exceed those achieved with monomeric subunit vaccines (29).

Building upon this technological advancement, the objective of the present study is to develop a novel, high-efficiency subunit vaccine against TGEV by leveraging the self-assembling properties of Ferritin (22). Herein, we describe the design and expression of a fusion protein comprising the TGEV S-RBD and the Ferritin nanoparticles. Furthermore, we evaluate the structural integrity of these assembled nanoparticles. Ultimately, this research seeks to provide a robust new strategy for the prevention and control of porcine transmissible gastroenteritis.

## 2 Materials and methods

### 2.1 Cell, plasmid, and reagents

Human embryonic kidney 293T (HEK293T) cells were maintained in our laboratory and cultured in Dulbecco’s Modified Eagle Medium (DMEM) supplemented with 10% fetal bovine serum (FBS) and 1% penicillin–streptomycin (100 U/mL penicillin, 100 μg/mL streptomycin), incubated at 37 °C with 5% CO₂. Chinese hamster ovary (CHO) cells were purchased and maintained in our laboratory, cultured in CHO Grow^®^ CD transient transfection serum-free medium, and incubated at 37 °C with 8% CO₂. The eukaryotic expression vector pCAGGS plasmid was preserved in our laboratory. The lentiviral vector pLVX-IRES-ZsGreen1 and packaging helper plasmids psPAX2, pMD2.G were also preserved in our laboratory.

### 2.2 Eukaryotic expression plasmid construction

**Figure 1:**
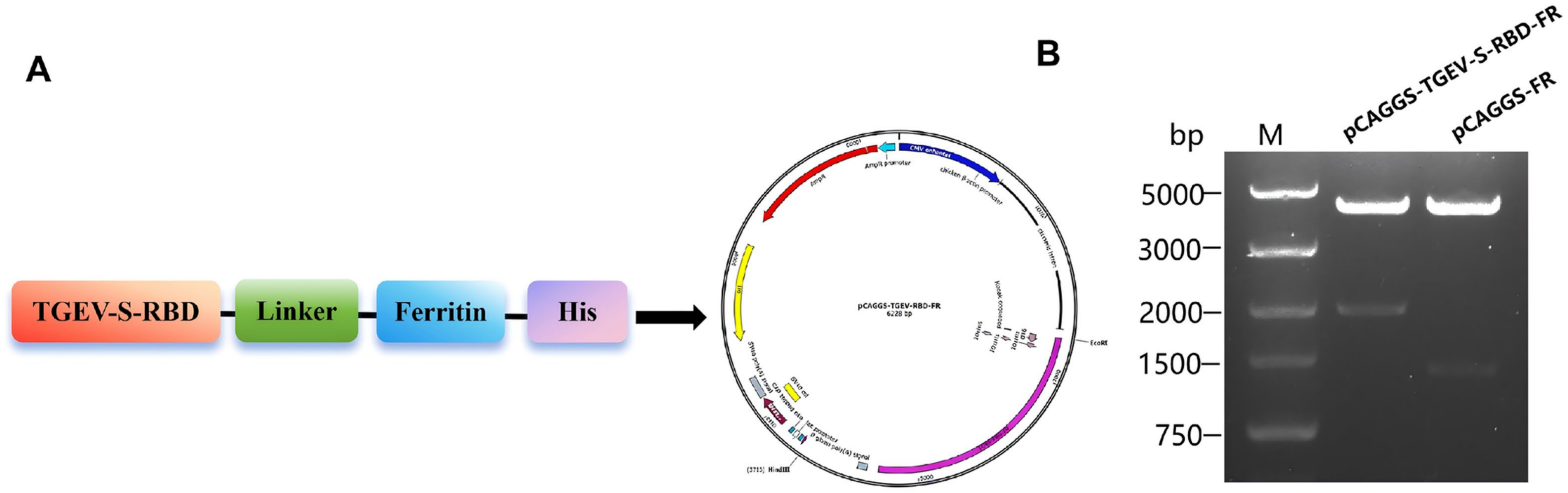
Construction and characterization of ferritin-based TGEV-S-RBD nanoparticles. **(A)** Schematic representation of the TGEV-S-RBD-FR nanoparticle design. **(B)** Restriction enzyme analysis of recombinant pCAGGS expression plasmids. Lanes show plasmids before and after double digestion at EcoRI and HindIII sites to confirm insert integration.

The S-RBD of TGEV was fused to the N-terminus of ferritin (FR) via a GGCGGGGGCGGCAGC flexible linker. A Kozak sequence (GCCACCAUGG), a GT6 signal peptide (MGWSCIILFLVATATGVHS), and a His-tag (HHHHHHHHHH) were placed at the N-terminus of the S-RBD. An *Eco*RI site was inserted at the 5′ end, and a HindIII site was inserted at the 3′ end. The empty pCAGGS vector was double-digested with *EcoR*I and *Hind*III, and the linearized vector and target fragments (TGEV-S-RBD and FR) were recovered using a gel extraction kit. The recovered pCAGGS vector was mixed with the TGEV-S-RBD and FR fragments, ligated overnight at 16 °C with T4 DNA ligase, and transformed into *Escherichia coli* DH5α competent cells. Transformants were plated on ampicillin LB agar, single colonies were cultured, plasmids were extracted by mini-prep, and the constructs were validated by Sanger sequencing and restriction digestion. Correctly verified eukaryotic expression plasmids were designated as pCAGGS-TGEV-S-RBD-FR and pCAGGS-FR (Figure 1A).

### 2.3 Expression and identification of recombinant proteins

HEK293T cells were removed from −80 °C storage and immediately thawed in a 37 °C water bath. Cells were transferred into DMEM supplemented with 10% fetal bovine serum and 1% penicillin–streptomycin and maintained at 37 °C with 5% CO_2_. Cells were seeded in six-well plates 1 day before transfection. At 80–90% confluence, the pCAGGS-TGEV-S-RBD-FR and pCAGGS-FR expression plasmids were transfected into HEK293T cells using X-treme GENE HP DNA transfection reagent at a ratio of plasmid to transfection reagent of 1:2. Following 48 h incubation at 37 °C, culture supernatants and cell pellets were collected separately.

Cell pellets were lysed and sonicated, and protein expression was subsequently verified via SDS-PAGE and Western blotting. Protein samples were mixed with 6 × SDS loading buffer and denatured at 100 °C for 10 min. Following centrifugation, the supernatants were resolved by electrophoresis. The protein was transferred onto PVDF membranes using an ice-cold electrotransfer system. The membranes were rinsed with PBST, blocked with QuickBlock™ buffer for 20 min at room temperature, and then incubated with an HRP-conjugated mouse anti-His-tag monoclonal antibody (1:3,000) for 2 h at room temperature. Finally, target proteins were visualized using an ECL chemiluminescent substrate.

### 2.4 Construction of lentiviral packaging vectors and generation of recombinant lentiviruses

**Figure 2:**
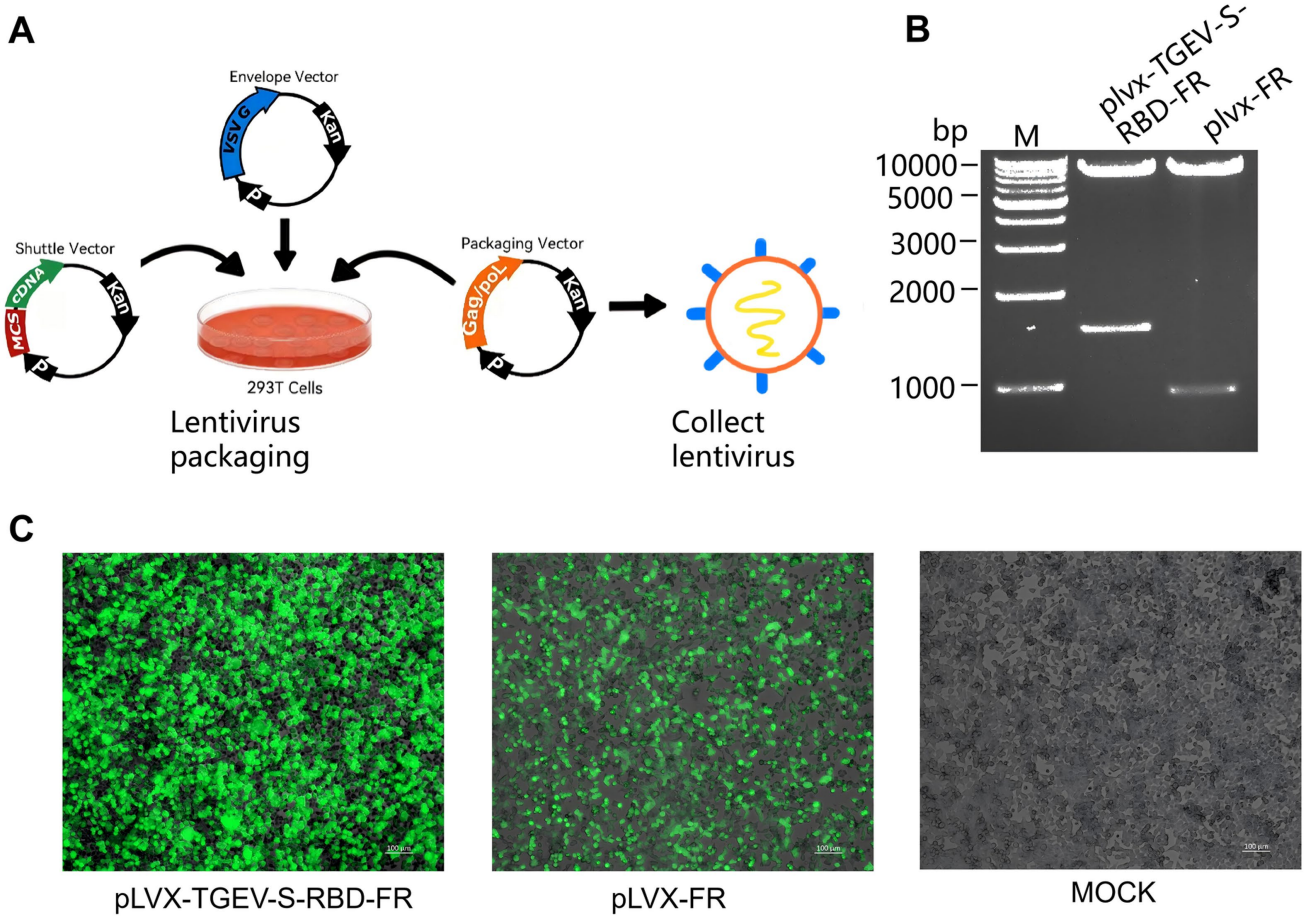
Development, verification, and functional characterization of recombinant lentiviral vectors. **(A)** Schematic representation of the lentiviral packaging system. Lentiviral particles were generated using a three-plasmid co-transfection system in HEK293T cells. The cells were transfected with a target shuttle plasmid and two auxiliary helper plasmids at a 3:2:1 ratio. Following a 48 h incubation period, the culture supernatant was harvested to collect the recombinant lentiviruses. **(B)** Restriction enzyme digestion and PCR verification of the shuttle plasmid. Validation of the lentiviral shuttle plasmids was performed via PCR and restriction fragment length analysis. The images demonstrate the shuttle plasmid profiles before and after double digestion at the EcoRI and BamHI sites, confirming successful vector construction. **(C)** Fluorescence-based assessment of packaging efficiency. To evaluate the efficiency of the packaging process, 293T cells were visualized via fluorescence microscopy 48 h post-transfection. The expression of the fluorescent reporter gene serves as an indicator of successful plasmid uptake and viral protein synthesis.

Lentiviral expression plasmids (pLVX-TGEV-S-RBD-FR and pLVX-FR) were constructed for the large-scale production of recombinant self-assembling nanoparticles. Cloning was performed as described in Section 2.2, and the resulting constructs were validated using Sanger sequencing and double-digestion analysis. Recombinant lentiviruses were produced by co-transfecting HEK293T cells at 80–90% confluence in 10-cm dishes. The shuttle plasmid, psPAX2, and pMD2.G were combined at a 3:2:1 mass ratio using PolyJet™ reagent (1:2 v/w ratio) in serum-free DMEM (Figure 2A). Following a 20-min incubation, the DNA-lipid complexes were added dropwise to the culture. The medium was replaced with fresh complete medium 6 h post-transfection. After 48 h, transfection efficiency was verified via fluorescence microscopy. The virus-containing supernatant was harvested, cleared of debris by centrifugation (6,000 rpm, 15 min, 4 °C) and 0.45 μm filtration, and subsequently concentrated 10-fold using ultrafiltration (4,500 ×g, 20 min, 4 °C) for titer determination.

### 2.5 Generation and purification of ferritin-based nanoparticle

**Figure 3:**
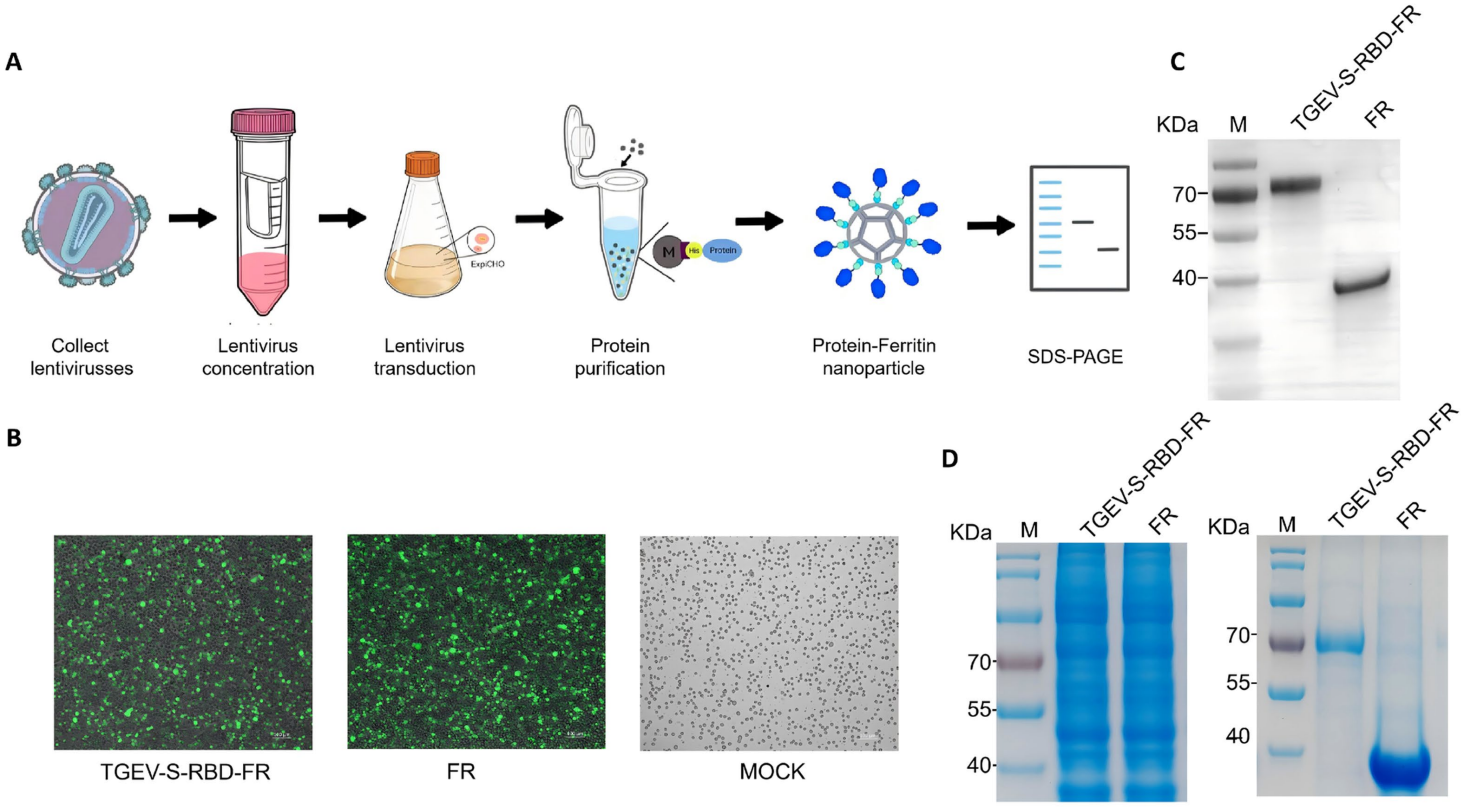
Stable expression, purification, and self-assembly of ferritin-based nanoparticle antigens. **(A)** Schematic workflow of antigen production and nanoparticle assembly. The experimental pipeline illustrates the concentration of recombinant lentiviral particles and the subsequent transduction of ExpiCHO cells at a multiplicity of infection (MOI) of 20. The secreted recombinant proteins were harvested and isolated using His-tag affinity purification with magnetic beads. The final stage depicts the *in vitro* self-assembly of purified subunits into spherical nanoparticles. **(B)** Assessment of lentiviral transduction efficiency. Transduction success in ExpiCHO cells was evaluated via fluorescence microscopy 48 h post-infection. The presence of reporter signals confirms stable integration and high-level expression within the host cell population. **(C)** Immunological validation of TGEV-S-RBD-FR. The identity and specificity of the purified fusion protein were verified by western blotting, confirming the presence of the TGEV-S-RBD-FR construct at the expected molecular weight. **(D)** Purity analysis of recombinant proteins. The efficiency of the purification process was assessed using SDS-PAGE. Lane analysis demonstrates the purity and integrity of the recombinant proteins harvested from the ExpiCHO culture supernatant prior to assembly.

ExpiCHO cells in the logarithmic growth phase (viability >95%) were transduced at a multiplicity of infection (MOI) of 20 in the presence of 8 μg/mL polybrene. Transduction efficiency was confirmed 48 h post-transduction via green fluorescence microscopy. To isolate the recombinant proteins, cells were harvested, and the resulting pellet was thoroughly solubilized in a lysis buffer containing 8 M urea. The lysate was incubated overnight at 4 °C with His-tag affinity magnetic beads. After washing with a low-concentration imidazole buffer to remove non-specific proteins, the target proteins were eluted using a high-concentration imidazole buffer. The purified nanoparticles were then analyzed by SDS-PAGE, as described in Section 2.3, to verify their purity and molecular weight (Figure 3A).

### 2.6 Characterization of nanoparticles

The purified protein was refolded and concentrated using a dialysis bag in refolding buffer (50 mM Tris–HCl, 150 mM NaCl, 400 mM L-arginine, 5% glycerol) with sequential urea concentrations of 6 M, 4 M, 2 M, 1 M, 0.5 M, and 0 M. The identity and integrity of nanoparticles were confirmed by Western blotting as described in Section 2.3. For ultrastructural characterization, carbon-coated copper grids were hydrophilized via glow discharge (15 mA, 60 s). A 4 μL sample aliquot was applied to each grid for 2 min, and excess liquid was removed with filter paper. The grids were then negatively stained with 3% uranyl acetate for 2 min, blotted, and air-dried for 15–20 min before imaging by transmission electron microscopy (TEM).

## 3 Result

### 3.1 Design and characterization of TGEV-S-RBD-FR constructs

The eukaryotic expression plasmids (pCAGGS-TGEV-S-RBD-FR and pCAGGS*-*FR) were validated via Sanger sequencing and restriction analysis using EcoRI and HindIII. Double digestion liberated the expected fragments for TGEV-S-RBD-FR (~1,900 bp) and FR (~1,408 bp) from the 4,244 bp pCAGGS backbone, consistent with theoretical predictions (Figure 1B).

**Figure 4:**
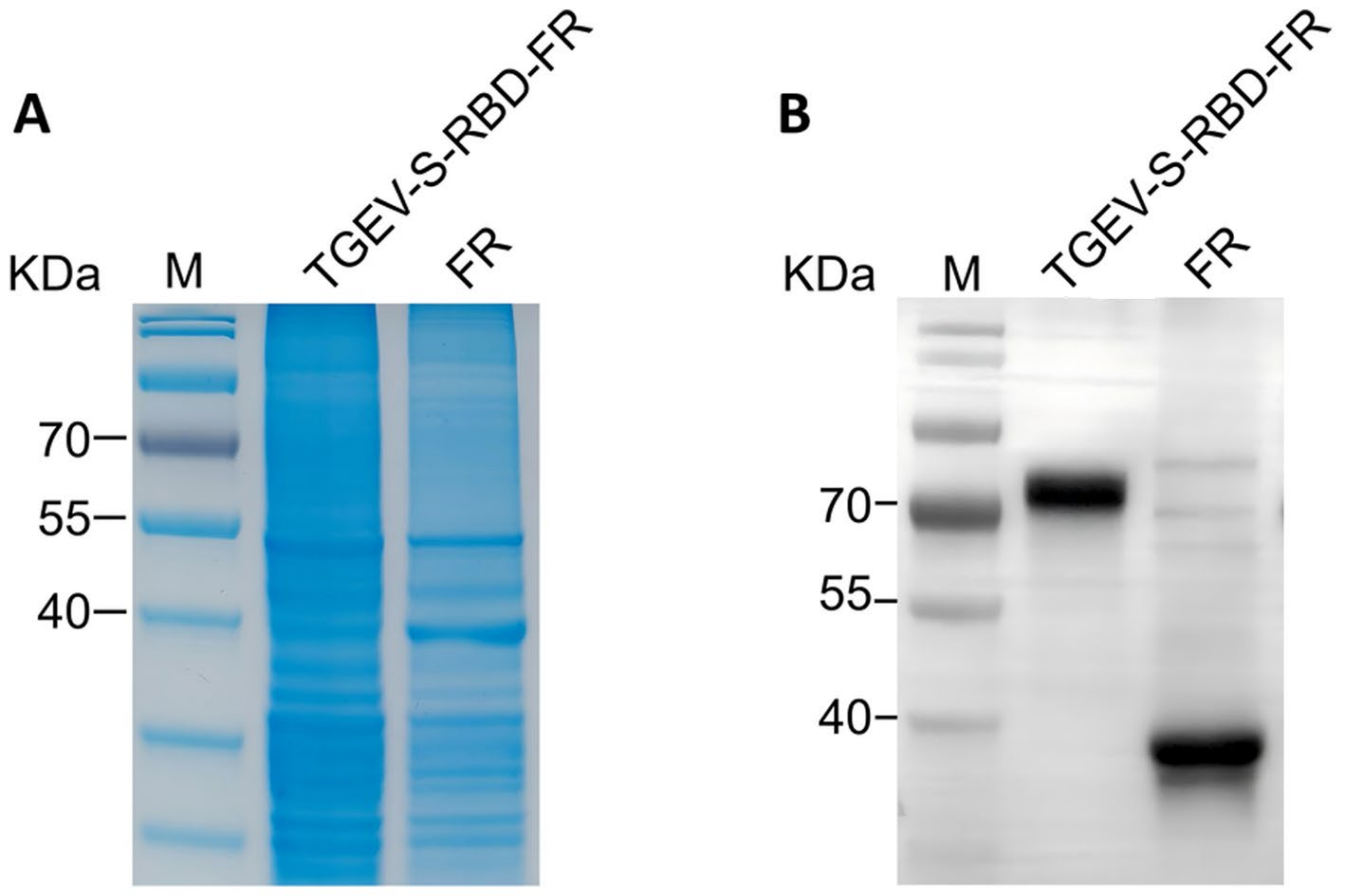
Expression and validation of ferritin-based nanoparticles in 293T cells. Recombinant proteins were analyzed 48 h post-transfection. **(A)** SDS-PAGE analysis of cell lysates; **(B)** Western blotting confirmation using anti-His specific antibody. Labeled bands correspond to the predicted molecular weights of TGEV-S-RBD-FR (~72 kDa) and the FR scaffold control (~40 kDa).

Following large-scale preparation, the sequence-verified plasmids were transfected into HEK293T cells. At 48 h post-transfection, protein expression was analyzed via SDS-PAGE (Figure 4A) and Western blotting (Figure 4B). As illustrated in Figure 4, both TGEV-S-RBD-FR and FR were successfully detected at their predicted molecular weights of approximately 72 kDa and 40 kDa, respectively, confirming the robust expression of the target antigens in a mammalian system.

### 3.2 Generation and titration of recombinant lentiviruses

The lentiviral shuttle plasmids were verified via restriction digestion with EcoRI and BamHI. Digestion yielded the anticipated fragments of ~1,442 bp and ~950 bp alongside the 8,172 bp pLVX-IRES-ZsGreen1 backbone, consistent with predicted sizes (Figure 2B). To produce recombinant lentiviruses, the shuttle plasmid was co-transfected with the packaging and envelope plasmids (psPAX2 and pMD2.G) into HEK293T cells. After 48 h of incubation, robust ZsGreen1 expression was observed via fluorescence microscopy, confirming high transfection efficiency and successful viral packaging (Figure 2C).

### 3.3 Expression and purification of recombinant ferritin fusion proteins

To produce the vaccine candidates, ExpiCHO cells were transduced with recombinant lentivirus at an MOI of 20. Robust green fluorescence at 48 h post-transduction confirmed high transduction efficiency and viral viability (Figure 3B). SDS-PAGE and Western blotting analysis of the cell lysates verified the expression of TGEV-S-RBD-FR and FR. Following large-scale culture, the proteins were isolated via His-tag affinity purification. The purified products yielded distinct bands at approximately 72 kDa and 40 kDa, respectively, which were consistent with the predicted molecular weights and confirmed by Western blotting (Figures 3C,D).

### 3.4 *In vitro* assembly and morphological characterization of nanoparticles

**Figure 5:**
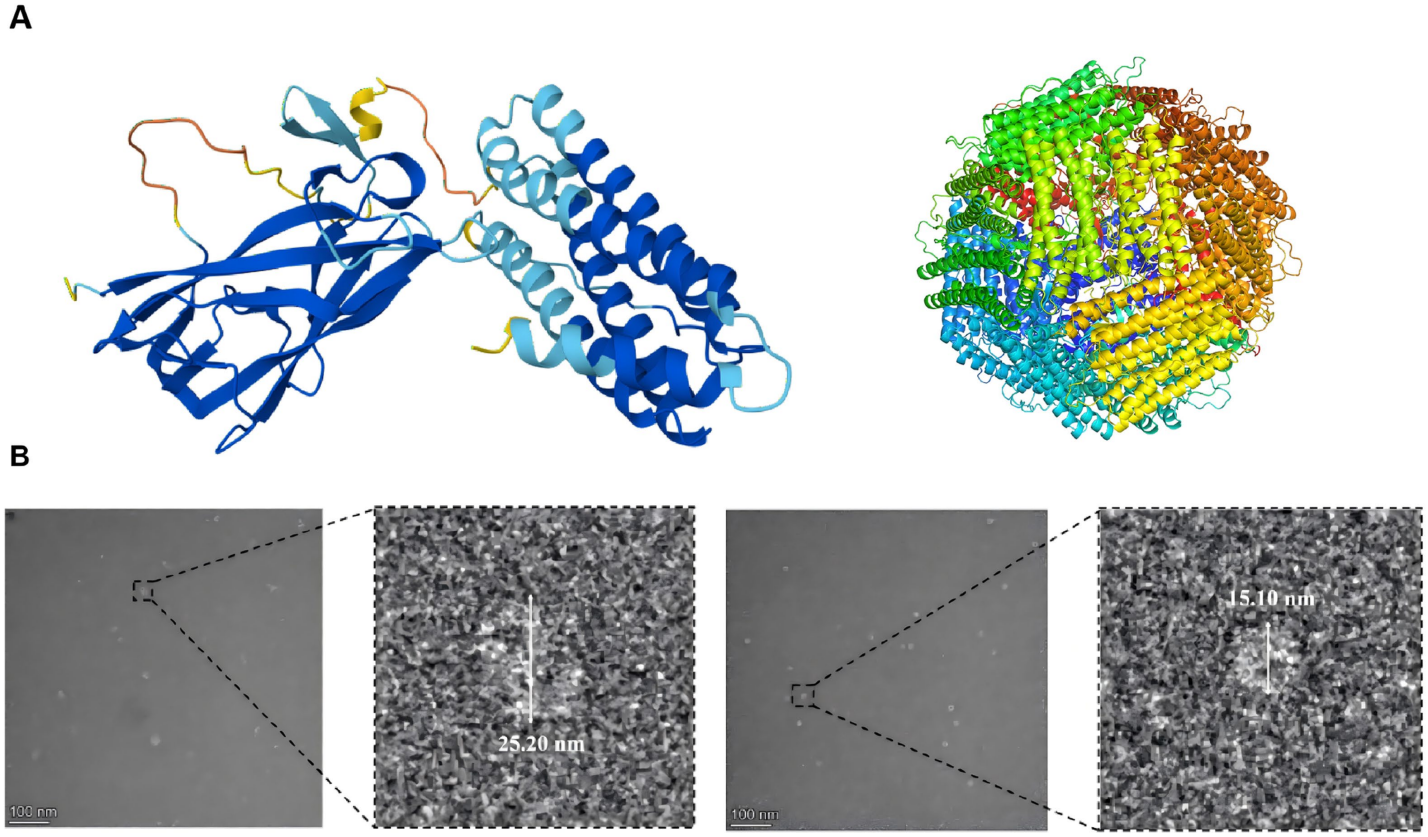
Structural design and ultrastructural morphology of TGEV-S-RBD-FR and ferritin nanoparticles. **(A)** Schematic representation of the recombinant constructs. The architectural layout of the 6 × His-tagged TGEV-S-RBD fusion protein (left) and the wild-type ferritin (FR) scaffold (right) are shown. These diagrams illustrate the genetic fusion of the viral receptor-binding domain (RBD) to the N-terminus of the ferritin subunit to facilitate surface display. **(B)** Morphological characterization via transmission electron microscopy (TEM). Representative TEM images of negatively stained nanoparticles are shown to verify self-assembly. Left: TGEV-S-RBD-FR nanoparticles displaying the S-protein RBD on the ferritin core. Right: Bare recombinant ferritin particles serving as the structural control.

To develop a nanoparticle-based vaccine candidate, we designed a chimeric construct where the TGEV-S-RBD was genetically fused to the N-terminus of the FR subunit, preceded by a 6 × His-tag for purification. This architectural layout was engineered to exploit the self-assembling properties of the ferritin 24-mer nanoparticles to display the viral RBD on the external surface (Figure 5A). TEM micrographs of negatively stained samples confirmed that both the TGEV-S-RBD-FR fusion proteins and the wild-type FR scaffolds successfully assembled into monodisperse, spherical nanoparticles (Figure 5B). Both variants exhibited the characteristic hollow-core morphology expected of successfully formed nanoparticles. The structural integrity and self-assembly of the resulting recombinant proteins were assessed using transmission electron microscopy (TEM) and particle size analysis. Transmission electron microscopy measurements revealed differences in the core diameters between the two samples. The TGEV-S-RBD-FR nanoparticles measured approximately 25 nm, whereas the bare FR scaffolds measured 15 nm. This significant increase in diameter is consistent with the projected external display of the S-RBD domains on the ferritin surface. These results collectively confirm that the recombinant fusion proteins maintain their self-assembly capabilities and successfully form the target subunit nanoparticles.

## 4 Discussion

This study successfully demonstrates the feasibility of a self-assembling ferritin-based nanoparticle platform for the multivalent display of the TGEV-S-RBD antigen. Our characterization data confirms that the fusion proteins (72 kDa for TGEV-S-RBD-FR and 40 kDa for the scaffold) assemble into monodisperse, spherical nanoparticles. Notably, the observed increase in particle diameter from 15 nm to 25 nm serves as direct evidence of successful antigen presentation on the nanoparticle’s surface. This structural transition validates our design strategy. By leveraging the ferritin scaffold, it enables high-density display of the key antigen (S-RBD) on the surface of ferritin nanoparticles, thereby better mimicking the native structure of the virus, providing a sophisticated foundation for vaccine development.

Beyond structural assembly, the strategic isolation of the S-RBD offers distinct immunological advantages (30–32). By focusing on the RBD, we minimize non-neutralizing epitopes, thereby reducing the risk of off-target responses or antibody-dependent enhancement (ADE) often associated with full-length Spike proteins (30). The use of the ExpiCHO expression system further ensures that critical post-translational modifications, such as N-glycosylation and disulfide bond formation, are preserved for optimal ACE2 receptor recognition (33). Crucially, this platform transforms monomeric RBD into highly organized 24-mer nanoparticles. This high-density presentation facilitates potent B-cell receptor cross-linking, while the 20–50 nm size range is evolutionarily optimized for rapid lymphatic trafficking. Additionally, the inherent robustness of the ferritin core provides superior stability against pH and thermal fluctuations, which is vital for maintaining efficacy in diverse field conditions (34–36).

The necessity for such a platform is underscored by the fundamental drawbacks of current TGEV vaccination strategies. While live-attenuated vaccines are immunogenic, they carry the inherent risk of reversion to virulence or genetic recombination with wild-type strains, a significant concern in neonatal swine populations (37). Conversely, inactivated vaccines offer improved safety but often suffer from suboptimal immunogenicity and a failure to trigger the robust mucosal responses required for enteric protection (22). Our ferritin-based approach bridges this gap; it provides the safety profile of a subunit vaccine while achieving the potent immunogenicity typically reserved for live-attenuated platforms. Consequently, this technology may reduce the frequency of booster administrations, alleviating the economic and logistical burdens on swine producers.

Despite these promising results, several technical challenges must be addressed before clinical application. Current findings are primarily limited by a lack of *in vivo* data; the protective efficacy and actual immune kinetics in live swine remain theoretical. Furthermore, the scalability of the lentiviral production system and the long-term stability of the formulation under various storage conditions require further investigation. Future research will therefore prioritize comprehensive immunological evaluations, moving from *in vitro* B-cell activation assays to controlled swine trials. These studies will focus on quantifying neutralizing antibody titers and assessing protection against live TGEV challenges. Additionally, we aim to optimize bioprocessing and purification strategies to ensure commercial viability. Given the modular nature of the ferritin scaffold, this platform also holds significant potential for rapid adaptation to other emerging veterinary coronaviruses, such as PEDV and PDCoV.

## 5 Conclusion

This research successfully developed a novel, biomimetic nanoparticle vaccine platform by leveraging the self-assembling properties of ferritin to achieve a high-density, 24-mer display of the TGEV Spike receptor-binding domain (S-RBD). By transforming the typically low-immunogenic monomeric RBD into a 25-nm organized structure, this platform bridges the critical gap between vaccine safety and potency: isolating the RBD eliminates risks of reversion to virulence or antibody-dependent enhancement (ADE), while the multivalent architecture facilitates high-avidity B-cell engagement and optimized lymph node trafficking. Furthermore, the use of a lentiviral-transduced ExpiCHO system ensures the authentic post-translational modifications and industrial scalability necessary for high-fidelity production. Ultimately, this ferritin-based scaffold establishes a framework that is not only a promising strategy for TGEV control but is also rapidly adaptable to other devastating porcine coronaviruses like PEDV and PDCoV.

## Data Availability

The raw data supporting the conclusions of this article will be made available by the authors, without undue reservation.
